# Cough medicine prescriptions for children by physician specialty and healthcare sector: a Finnish population-based nationwide register study

**DOI:** 10.1007/s00431-025-06306-2

**Published:** 2025-07-08

**Authors:** Péter Csonka, Fredriikka Nurminen, Eero Lauhkonen, Heini Kari

**Affiliations:** 1 Tampere Center for Child, Adolescent and Maternal Health Research, Faculty of Medicine and Health Technology, Tampere University, Arvo Ylpönkatu 34, 33520 Tampere and Terveystalo Healthcare, Rautatienkatu 27, 33100, Tampere, Finland; 2 Research Unit, The Social Insurance Institution of Finland (Kela), Helsinki, Finland; 3 Tampere University and University Hospital, Tampere, Finland

**Keywords:** Cough and cold medicines, Children, Prescribing, Register study

## Abstract

Despite clear clinical guidelines advising against the use of cough and cold medicines (CCMs) in children, these products continue to be prescribed. We aimed at describing recent trends and identifying deviations from guidelines to inform future targeted interventions. We retrospectively retrieved data on all CCM prescriptions for children under 16 years of age in Finland between 2017 and 2023 from the centralised nationwide prescription registry. Descriptive statistical methods were used to analyse the data, stratified by child’s age, CCM type, physician’s employment sector, and medical specialty. The dataset included 96,499 prescriptions for 75,281 children. Overall, CCM prescribing decreased significantly from 2017 to 2023, particularly between 2017 and 2020, with the steepest decline in the 2–4.99-year age group. Prescribing levels did not return to pre-pandemic levels in any age group. Opium alkaloids and their derivatives were the most commonly prescribed CCMs for children under the age of 12. CCM prescribing rates were nearly identical across public and private sectors. Approximately 40% of prescriptions were issued by non-specialists and 18% by medical students—who constituted the largest group of public sector prescribers. In the private sector, the most frequent prescribers were paediatricians, non-specialists, general medicine specialists, and otolaryngologists. *Conclusions*: CCMs and especially opium alkaloids and their derivatives are still being prescribed even to small children. These findings should prompt nationwide multifaceted clinical guidance campaigns and interventions targeting medical students and non-specialist physicians, as well as private sector providers, including paediatricians and other specialists.

**Table:** 

**What is known:** • *Despite guidelines advising against cough and cold medicines (CCMs) in children, prescribing persists*.	
**What is new:** • *CCM prescriptions for children declined markedly in Finland between 2017–2023, however opium alkaloids remained the most common among children under 12, despite CNS safety concerns. Non-specialists, general practitioners, and medical students prescribed CCMs most frequently. Specialist physicians were more likely to prescribe CCMs in the private sector, while the public sector had a broader mix of prescribers*.

**Supplementary Information:**

The online version contains supplementary material available at 10.1007/s00431-025-06306-2.

## Introduction

Cough is a common reason for medical consultations in children, often driven by parental concerns about sleep disturbances and potential complications from respiratory infections [1, 2]. Cough and cold medicines (CCMs), historically prescribed to alleviate symptoms, have raised increasing concerns due to their questionable efficacy and potential for adverse effects [3–11]. CCMs containing codeine or hydrocodone are also associated with misuse and addiction in adolescents and with potentially fatal respiratory depression in young children [12, 13].

Regulatory bodies such as the European Medicines Agency (EMA), U.S. Food and Drug Administration (FDA), and Health Canada have issued warnings against the use of these medications in children due to safety concerns [8, 9, 14]. Similarly, medical guidelines, including the Finnish Current Care Guidelines, discourage the use of CCMs in paediatric populations, especially for children under 6 years of age [5, 15].

Despite these recommendations, the changes in CCM prescribing practices have been relatively limited and slow [16–18], and physicians still prescribe CCMs even for preschool children [16, 19]. A recent study conducted in Finland demonstrated that systematic interventions, which included the distribution of educational materials and real-time prescription monitoring, significantly reduced cough medicine prescriptions in paediatric populations [20, 21]. However, this intervention was limited to one private healthcare company covering only about 10% of outpatient paediatric visits. A more comprehensive nationwide analysis is needed, including both public and private healthcare services, to identify possible deviations from current care guidelines.

This article aims to explore trends in CCM prescriptions for the entire Finnish paediatric population, focusing on variations across different age groups, types of medicines, and differences between public and private healthcare sectors. We also examine the influence of physician specialty on prescribing patterns, providing insights into how targeted interventions can bridge the gap between clinical recommendations and real-world practice, ultimately leading to safer and guideline-concordant management of paediatric cough.

## Methods

This was a nationwide retrospective register-based study. The data were collected from the Prescription Centre, which is a centralised national database of prescription and dispensation data in Kanta Services. The Prescription Centre includes all prescriptions issued for the outpatient setting and their dispensations from community pharmacies [22].

For this study, we retrieved data from the Prescription Centre on all oral prescriptions in the Anatomical Therapeutic Chemical (ATC) Classification Index class R05 (cough and cold preparations, CCMs) and its chemical substance subgroups prescribed between 2017 and 2023, excluding R05CB13 (Dornase alfa for cystic fibrosis). For each prescription, data on the patient's birth date, specifics of the prescribed medication, and details regarding the physician’s medical specialty and employment sector were collected when available. Prior to analyses, cancelled prescriptions and individuals without a personal identification code were excluded from the data. Information on the physician’s specialty was unavailable for 5.9% of all prescriptions, and in 2023, this information was missing for 3.7% of prescriptions. Data on the employment sector were unavailable for less than 0.01%. The study population included all children aged 0–15 in Finland to whom CCMs had been prescribed for outpatient care during the study period.

The patient’s age was defined as the age at the end of each year. Descriptive methods were used to study the yearly rates of CCMs per 1000 persons during the study period. Subgroup analyses for children’s age groups (< 2 years, 2–4.99 years, 5–11.99 years, and 12–15.99 years) were performed to describe the most commonly prescribed CCMs in each age group. Furthermore, the numbers of prescriptions were analysed by physicians’ employment sector and medical specialties. We collected data on all prescriptions; hence, no formal sample size calculation was needed. Data processing and analysis were conducted using R statistical computing software (version 4.3.3).

As the study used secondary register data, ethics board approval was not required under Finnish law. The Social Insurance Institution of Finland (Kela) authorised the data use. The person-level data on prescriptions and dispensed medications used in the study were fully pseudonymised before the authors accessed the data. All data preparation and linkage in the study were done with pseudo-identifiers. The authors did not have access to information that could identify individual participants at any stage of the study.

## Results

**Fig. 1:**
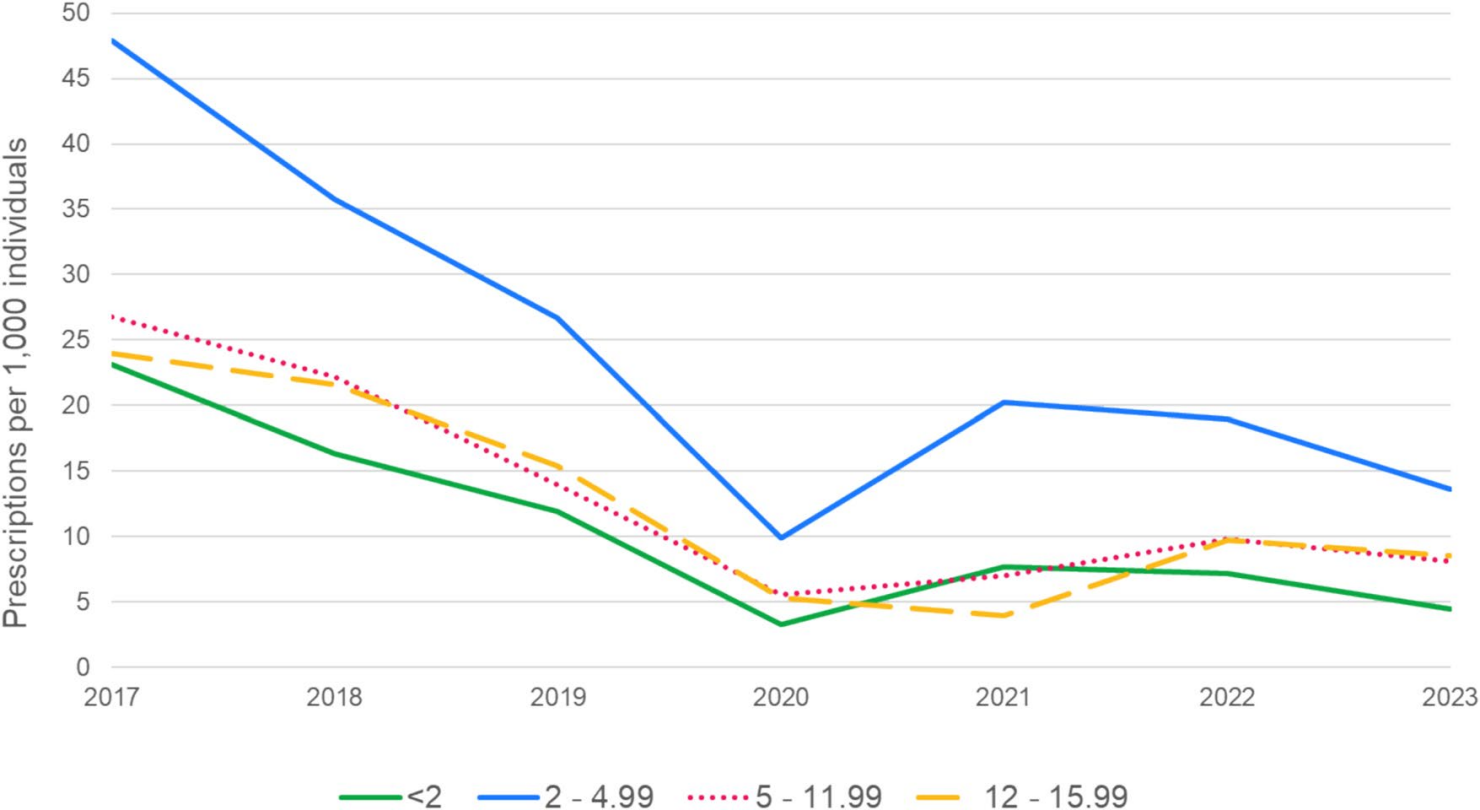
Annual rates of cough and cold medicine prescriptions per 1000 children younger than 16 years, stratified by age groups, from 2017 to 2023

A total of 96,499 prescriptions were issued for 75,281 children during the study period. The number of CCM prescriptions per 1000 individuals for children under the age of 16 was highest in 2017 across all age groups (Fig. 1). There was a declining trend in CCM prescribing rates during the years preceding the COVID-19 pandemic. There was a sharp reduction in the prescription rates in 2020, likely resulting from decreased respiratory tract infections during the social distancing measures [23]. The lowest number of CCM prescriptions was recorded in 2020 for children under 12 years. However, for children aged 12–15.99 years, the lowest annual rate (3.9 prescriptions per 1000 individuals) was recorded in 2021. The most significant reduction between 2017 and 2020 was observed in children aged 2–4 years, with the annual prescription rate decreasing from 48.3 to 10.0 per 1,000 individuals. By the end of 2023, none of the age groups had returned to the pre-pandemic annual rate of CCM prescriptions.

**Fig. 2:**
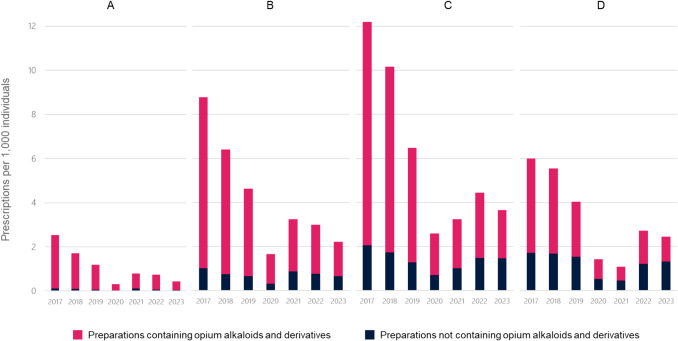
Annual rate of cough and cold medicine prescriptions per 1000 children younger than 16 years, stratified by age groups, from 2017 to 2023. **A** Children aged < 2 years. **B** Children aged 2–4.99 years. **C** Children aged 5–11.99 years. **D** Children aged 12–15.99 years

Table 1 demonstrates the number of CCM oral prescriptions for the R05 category from 2017 to 2023, stratified by active ingredients. Dextromethorphan/ephedrine combination was the most prescribed preparation every year. Ethylmorphine was the second most prescribed CCM from 2017 to 2019. However, beginning in 2020, the mucolytic agent ambroxol advanced from third to second place in prescription frequency. Age-group-level analysis indicates that this trend was predominantly driven by prescriptions for children aged 5–11.99 years (Fig. 2), among whom ambroxol became the most commonly prescribed CCM in 2023 (2.3/1000). Each year, preparations containing opium alkaloids and their derivatives were the most frequently prescribed CCMs across all age groups (Fig. 2). The use of opium derivatives was most prevalent in the < 2-year age group, where 90.6% of the prescribed CCMs were opium derivatives still in 2023. The proportion of opium derivatives use in the older age groups declined from 2017 to 2023 as follows: in age group 2–4.99 from 88.6% to 71.3%; in age group 5–11.99 from 83.2% to 60.0%; and in age group 12–15.99 from 71.5% to 60.6%.

**Table 1:** Annual rate of cough and cold medicine prescriptions per 1,000 children younger than 16 years from 2017 to 2023

	**2017**	**2018**	**2019**	**2020**	**2021**	**2022**	**2023**
Expectorants and mycolytics, excluding combinations (R05C)							
Ambroxol^†^	3.74	3.15	2.75	1.24	1.7	2.62	2.54
Bromhexine^†^	0.66	0.56	0.38	0.15	0.47	0.41	0.32
Carbocisteine^†^	0.03	0.02	0.02	0.01	0.02	0.02	0.01
Erdosteine	0.13	0.14	0.12	0.04	0.04	0.15	0.19
Guaifenesin^†^	0.08	0.09	0.02	0	0	0	0
N-acetylcysteine^†^	0.17	0.2	0.16	0.07	0.06	0.18	0.16
all R05C	4.81	4.17	3.46	1.51	2.3	3.4	3.22
Cough suppressants, excluding combinations (R05D)							
Dextromethorphan*^†^	0.40	0.29	0.28	0.19	0.40	0.63	0.50
Ethylmorphine*	9.05	7.65	4.37	0.75	0.54	1.10	0.76
Levodropropizine	0	0	0	0	0	0	0.18
Pentoxyverine^†^	0.02	0.02	0.02	0.02	0.11	0.06	0.02
all R05D	9.48	7.96	4.66	0.96	1.05	1.79	1.46
Cough suppressant and expectorant combinations (R05F)							
Codeine* + guaifenesin^†^	0.02	0.01	0	0	0	0.01	0.01
Dextromethorphan* + ephedrine	13.23	10.22	7.6	3.45	4.95	5.66	4.05
Codeine + ephedrine + diphenhydramine	1.95	1.44	0.57	0.04	0.03	0	0
Others	0.04	0.04	0.04	0.02	0.02	0.04	0.02
all R05F	15.24	11.7	8.21	3.51	4.99	5.72	4.09

Ammonium chloride and hydrocodone preparations are not available in Finland

*Opium alkaloids and derivatives

^†^Also available over-the-counter

**Fig. 3:**
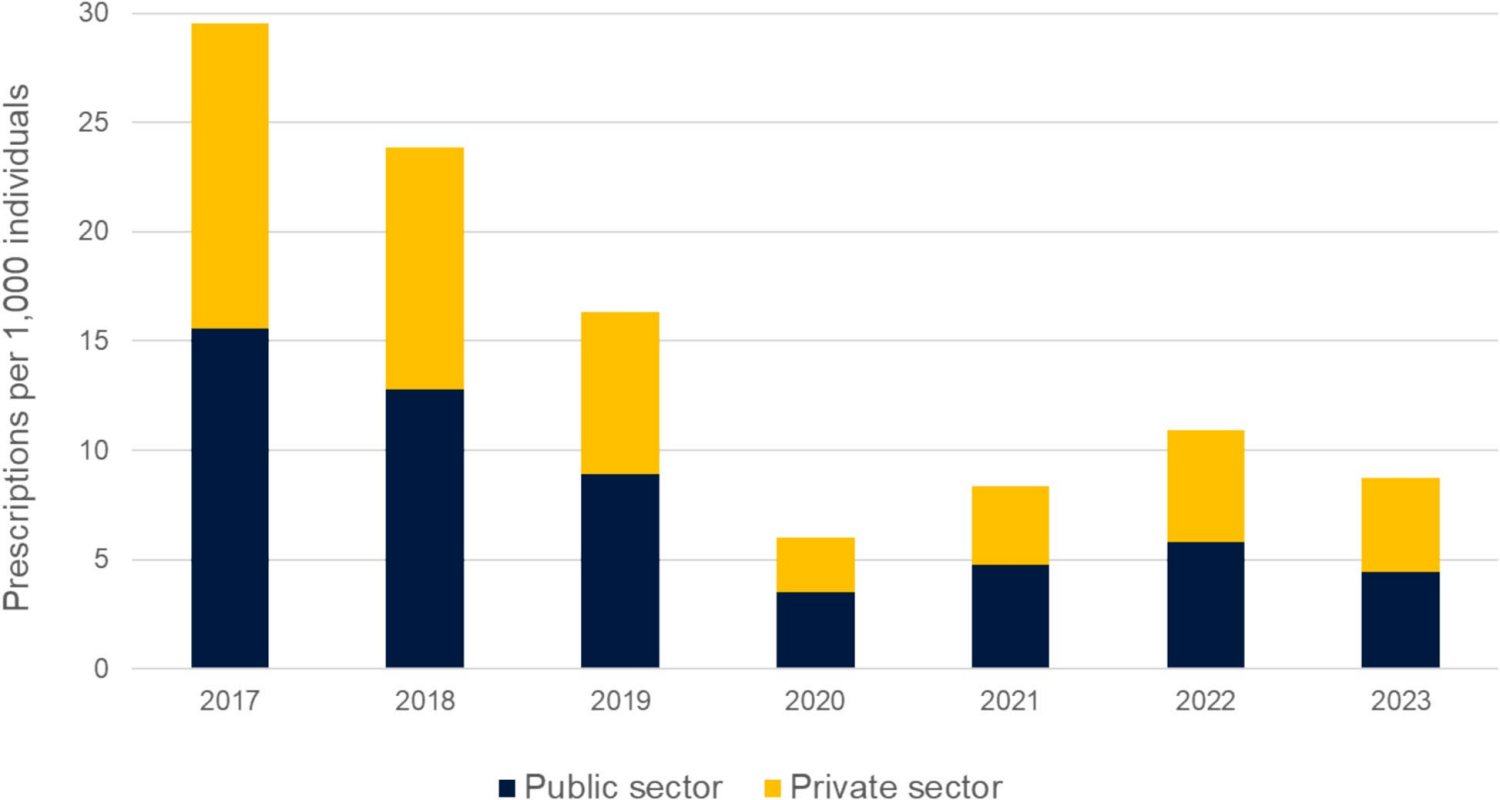
Annual rate of cough and cold medicine prescriptions per 1000 individuals for children younger than 16 years, stratified by the physician’s working sector, from 2017 to 2023. Data on the employment sector were unavailable for less than 0.01%

Figure 3 illustrates the annual rate of CCM prescriptions for children younger than 16 years, categorised by the physician’s working sector, from 2017 to 2023. The contribution of the private sector has slightly increased from 47.3% in 2017, though settling to a somewhat balanced distribution of prescriptions between private (49.1%) and public (50.9%) sector physicians in 2023.

**Fig. 4:**
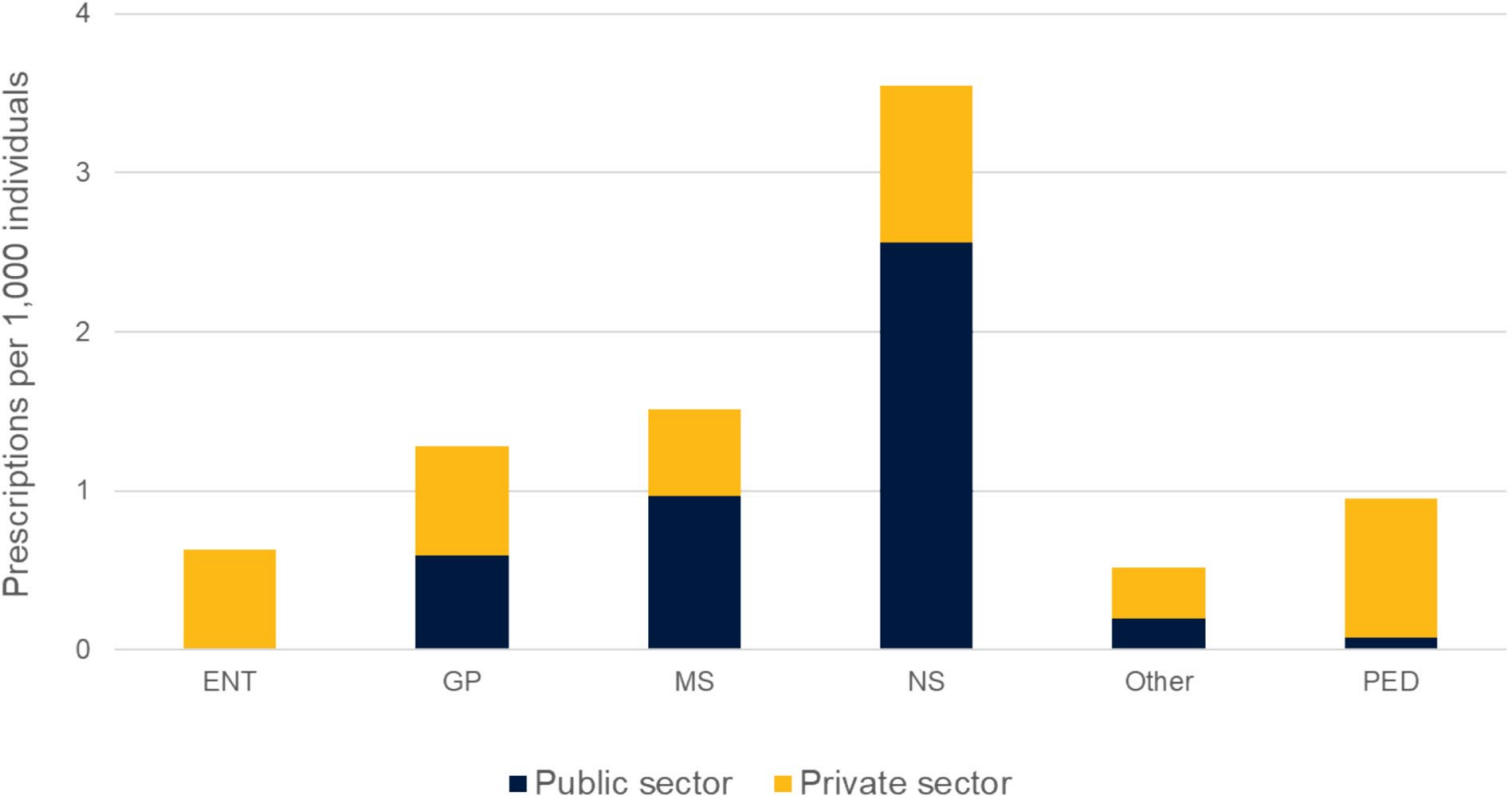
Number of cough and cold medicine prescriptions for children younger than 16 years, stratified by physicians’ medical specialties and working sectors, during year 2023. Information on the physician’s specialty was unavailable for 5.9% of all prescriptions, and in 2023, this information was missing for 3.7% of prescriptions. Data on the employment sector were unavailable for less than 0.01%. ENT = Ear, Nose and Throat specialist, GP = General Practitioner (specialists in general medicine), MS = Medical Student, NS = Physician with no medical Specialty, PED = Paediatrician, Other = all other specialists

The proportion and distribution of CCM prescriptions among different specialists were almost identical throughout the seven study years; hence, for clearer presentations, only year 2023 is illustrated in Fig. 4. Approximately 40% of children’s CCM prescriptions were issued by physicians without a medical specialty and 18% by medical students, representing the largest proportion of public sector physicians prescribing CCMs. In the private sector, the largest groups of physicians prescribing CCMs for children included paediatricians, physicians without a medical specialty, specialists in general medicine (GP), and otolaryngologists (ENT doctors). The Finnish primary healthcare and medical training system is explained in the online repository.

## Discussion

This comprehensive analysis of the Finnish national prescription database, including 96,499 CCM prescriptions, showed several up-to-date key findings. Most notably, the overall prescription frequency of CCMs for children declined significantly over the seven-year observation period, particularly between 2017 and 2020, and most prominently in the 2–4.99-year age group. Importantly, pre-pandemic levels of CCM prescribing were not reached again in any age group, suggesting a sustained shift in prescribing behaviour. This trend likely reflects the synergistic effect of evolving clinical guidelines, national and international regulatory measures, and targeted educational interventions.

Despite the overall decline, opium alkaloids and their derivatives remained the most frequently prescribed CCMs for children under the age of 12 throughout the study period—a concerning trend given the increased vulnerability of this age group to central nervous system effects. Ethylmorphine was the second most commonly prescribed CCM between 2017 and 2019. However, its prescribing dropped markedly following a regulatory warning issued in October 2019 advising against its use in children under the age of 12 [24]. This finding supports prior evidence suggesting that safety alerts and regulatory actions can have a rapid and significant effect on prescribing behaviour, often more impactful than clinical guidelines alone. For instance, in the USA, the withdrawal of over-the-counter (OTC) infant CCMs led to a substantial decline in emergency department visits for adverse events in children under the age of two [25].

International data support the effect of national level actions in safety alerts and regulatory advisory. An analysis of outpatient prescription data in Italy and the Netherlands between 2005 and 2008 revealed a modest decline in CCM prescribing in Italy (from 83 to 77/1000 children), while prescriptions increased in the Netherlands (from 74 to 92/1000), where no national warning had been issued at all [26]. In the USA, studies observed significant reductions in prescription CCM use following regulatory advisories between 2005 and 2010, although OTC use remained unchanged or increased in some settings [17, 19]. Similarly, in the UK, CCM prescribing among general practitioners declined by 84% between 1998 and 2018, reflecting increasing regulatory caution [27]. Canadian data also showed consistent declines in codeine prescribing across provinces after Health Canada advisories in 2013 and 2016, with adjusted level changes ranging from − 0.6% to − 18.4% and − 2.1% to − 17.9%, respectively [14].

In Finland, a particularly notable reduction in CCM prescriptions was observed following a phased intervention implemented by a nationwide private healthcare provider. Between 2017 and 2020, prescriptions dropped by over 90% among older children and were eliminated entirely in children under 2 years of age. This reduction was achieved through physician education, targeted electronic reminders, and personalised feedback [20, 21]. During the overlapping period (2017–2020), the study involving an active intervention showed both significantly lower CCM prescription rates and a faster decline than the national average observed in our study. These findings collectively highlight that while clinical guidelines and regulatory actions are essential, system-level interventions are often more effective in achieving real-world changes in prescribing behaviour.

We observed an increasing trend in the prescribing of ambroxol, which emerged as the most frequently prescribed CCM among children aged 12–15.99 years in 2023. At present, there is no definitive explanation for this shift, and the literature lacks specific data on the increased use of ambroxol in this age group. Given the limited evidence supporting ambroxol’s efficacy in treating acute cough in children, this prescribing pattern warrants further investigation.

Another concerning trend is the substitution of withdrawn or restricted CCMs with newer agents, often introduced through aggressive marketing. For example, levodropropizine has been increasingly promoted in Finland as a “safe” paediatric cough remedy, despite the lack of evidence for its efficacy in treating common cold symptoms in children. This underscores the persistent challenge of curbing inappropriate CCM use and highlights the need for stronger regulatory oversight and rigorous evidence-based evaluation before new products are widely adopted in clinical practice.

Although international research comparing CCM prescribing patterns between private and public healthcare sectors is limited, a study from Turkey found that CCMs were frequently prescribed across most healthcare settings except university hospitals [28]. In Finland, differences have been reported in antibiotic prescribing for paediatric lower respiratory tract infections between public and private providers [29], suggesting similar trends could exist for CCMs. However, our study found nearly identical overall CCM prescription rates in both sectors.

A more detailed analysis revealed important differences related to prescriber training and specialisation. Non-specialist physicians were the most frequent prescribers of CCMs, followed by general practitioners and medical students, indicating that clinical specialisation may be associated with greater adherence to paediatric evidence-based guidelines. Moreover, specialist physicians were more likely to prescribe CCMs in the private sector, while the public sector had a broader mix of prescribers. These patterns can be partly explained by the structural organisation of paediatric care in Finland. In the public sector, most acute respiratory infections are managed in outpatient settings by general practitioners, physicians in training, or non-specialised doctors, with only complex cases referred to specialist care at hospitals. In contrast, the private sector often provides more rapid access to outpatient care, including direct consultations with specialists such as paediatricians and ENT physicians. These systemic differences influence the case mix and likely contribute to variations in prescribing behaviour across sectors.

Our study has several strengths. This is the first comprehensive nationwide analysis of CCM prescriptions for children in Finland. We utilised a centralised national prescription registry covering the entire Finnish population, ensuring exhaustive population coverage and minimising selection bias. This validated registry reflects real-world clinical practice across both public and private healthcare sectors, and its standardised data recording enhances the reliability of our findings. To the best of our knowledge, our study is the first nationwide report on CCM prescriptions post-Covid.

Certain limitations must be acknowledged. The study does not capture OTC medication use or assess the appropriateness of prescriptions based on clinical indication or disease severity. It is also unknown whether prescribed medications were filled or taken as directed. In Finland, OTC purchases of CCM are reported as wholesale data for the entire population, without age-specific breakdowns. As a result, it is not possible to accurately assess or reliably estimate their use specifically among children. According to the Finnish Statistics on Medicines (Fimea), the defined daily dose per 1000 inhabitants per day for CCM declined from 5.46 in 2017 to 4.59 in 2023 [30]. Given this overall downward trend, it seems unlikely that OTC use increased during the same period in contrast to the observed decline in physician prescriptions. However, since OTC purchases are not recorded by age and may be used without medical consultation, this unmeasured usage could lead to a significant underestimation of the total medication burden. Acknowledging this limitation underscores the need for more comprehensive data sources to accurately assess paediatric exposure to cough medicines.

We did not adjust for provider-level or socioeconomic factors, nor did we evaluate broader healthcare outcomes. Children aged 16–18 were excluded due to variability in the transition to adult care in Finland, which could introduce bias. Additionally, the generalisability of our findings may be limited outside the Finnish healthcare context. Besides the R05 group, other medications, for example, salbutamol in oral solution, may be used off-label as a cough medication. Unlike the generally ineffective and harmful R05 substances, the R03 group is not subject to the same restrictions. The R03 substances have a well-established role in treating bronchial obstruction, which often presents with a cough as a symptom. In our study, we have focused solely on the R05 group to minimise bias. Nevertheless, our internal review of the prescribing data indicated no increase in oral bronchodilator use during the study period; these data are not presented in detail.

In conclusion, while clinical guidelines are a critical foundation for paediatric prescribing, our findings and international evidence suggest that a multifaceted approach is necessary to reduce inappropriate CCM use. Regulatory actions—including safety warnings, contraindications, and product labelling changes—have demonstrated significant impact, with market withdrawals being among the most effective interventions. Educational strategies should target medical students and non-specialist physicians, who are among the most frequent CCM prescribers, and should also encompass private sector providers, including specialists. Public education is equally important: empowering caregivers with accurate information about the limited benefits and potential harms of CCMs may reduce demand and alleviate the pressure to prescribe cough medicines for children. Finally, real-time implementation tools such as point-of-care alerts and clinical decision support systems, alongside continuous monitoring of prescribing trends and clinician benchmarking, can further promote evidence-based care for paediatric respiratory symptoms.

## Supplementary Information

Below is the link to the electronic supplementary material.

ESM 1 (DOCX 32.3 KB)

ESM 2 (DOCX 22.6 KB)

## Data Availability

No datasets were generated or analysed during the current study.
