# Supplementary material for: Cough medicine prescriptions for children by physician specialty and healthcare sector: a Finnish population-based nationwide register study
Source: Eur J Pediatr. 2025 Jul 8;184(8):471. doi: 10.1007/s00431-025-06306-2 (PMC12234626; doi:10.1007/s00431-025-06306-2)
Supplement: Supplementary file 1 — (DOCX 32.3 KB) [file 431_2025_6306_MOESM1_ESM.docx]

**Online repository supplementary information**

**Medical training in Finland explained**

In Finland, all medical specialists begin their careers by completing a university-level medical degree called the Licentiate of Medicine. This degree takes approximately 6 years and is offered at one of the five country’s medical faculties. It includes both theoretical and practical clinical training. After graduation, doctors complete a mandatory internship to obtain a license to practice medicine independently. Only then can they apply to specialist training programmes, each with its own duration, structure, and clinical focus.

As of the end of 2020, Finland had approximately 22,000 licensed doctors under the age of 65. Of these, around 13,000 were specialists, meaning that roughly 9,000 doctors had not completed specialist training. These non-specialist doctors include recent graduates as well as those still in training.

In public primary healthcare, which is mostly delivered through municipal health centres, specialists in general practice (similar to family physicians) account for about 30% of the medical workforce. The remaining 70% are typically either doctors still training to become specialists or non-specialised physicians, often early in their careers.

**General practitioners (specialist in general practice)**

In Finland, to become a general practitioner (GP)—formally recognised as a Specialist in General Practice—a doctor must complete a structured five-year specialist training programme following the Licentiate of Medicine degree. The programme begins with 9 months of full-time clinical work in primary healthcare, usually at a municipal health centre. It also includes at least a year of hospital-based training in relevant specialties such as internal medicine, paediatrics, psychiatry, or geriatrics. The majority of the training takes place in primary care settings, where doctors work under the supervision of experienced GPs. The focus is on developing the skills needed to provide comprehensive, continuous care for patients of all ages and across a wide range of medical issues. Trainees must also complete theoretical coursework and pass a final written examination before graduating.

**Ear, nose, and throat (ENT) specialists**

To become an ear, nose, and throat (ENT) specialist—formally titled a Specialist in Otorhinolaryngology in Finland—a doctor must complete a five- to six-year specialist training programme following the Licentiate of Medicine degree. The training begins with a minimum of 9 months in primary healthcare, typically in a public health centre, before continuing in hospital-based settings. The remainder of the programme is conducted in central and university hospitals, where doctors train in ENT departments and rotate through subspecialty areas such as otology (ear disorders), rhinology (nasal and sinus conditions), laryngology (voice and throat disorders), and head and neck surgery. The training combines hands-on surgical experience with structured theoretical education. Trainees must complete mandatory courses and pass a national written examination to demonstrate their competence.

**Paediatricians**

To become a paediatrician in Finland, a doctor must complete a six-year specialist training programme following the Licentiate of Medicine degree. The programme begins with at least 9 months of training in primary healthcare, usually in child health clinics or general practice settings, where doctors gain experience in preventive care and early childhood health. The remainder of the training takes place in paediatric departments within central and university hospitals, where doctors rotate through various areas of general paediatrics and paediatric subspecialties such as neonatology, paediatric emergency care, and paediatric cardiology. In addition to clinical training, the programme includes theoretical coursework and requires candidates to pass a national written examination.

**Physicians with no medical specialty**

In Finland, doctors who have completed the basic medical degree (Licentiate of Medicine) but have not specialised are fully licensed to practice medicine. Often referred to as non-specialist doctors or general physicians, they are authorised by Valvira, the national licensing authority, to work independently in a variety of clinical settings. These doctors typically work in health centres, hospitals, emergency departments, or occupational health services. Many are early in their careers and may work under the supervision of specialists or in multidisciplinary teams. Some take temporary or locum positions while gaining experience or considering future specialisation. Although they are not specialists in a specific field, they are trained to manage common medical issues and refer patients to specialised care when needed. While many eventually enter specialist training, others continue working as general physicians, especially in roles where broad clinical skills are in demand.

**Medical students working as doctors**

In Finland, a medical student who has completed 5 years of studies can apply for a temporary license. With this license, they are allowed to work as a GP in a health centre or hospital in specific roles. A student with this temporary license may, in some cases, also prescribe medications, but usually with certain restrictions and under the supervision of a senior physician.

**Primary healthcare system in Finland explained**

Finland’s primary care system is a central component of the country’s publicly funded healthcare system. It is built around municipality-run health centres that serve as the first point of contact for most medical issues. The system is designed to provide comprehensive, accessible, and equitable care to the population. Services are funded mainly through tax revenue and provided to residents at low cost.

In the Finnish primary care system, patients are treated by GPs, physicians specialising to be GPs, and doctors without any specialty. GPs provide preventive care, management of acute and chronic conditions, minor procedures, and referrals to specialised care when needed. Health centres also serve as training sites for medical students and doctors in specialist training (especially in general practice).

Acute common respiratory infections in children are primarily treated outside hospitals. Most visits are due to upper respiratory tract infections. In the public sector, infections treated outside the hospital are managed at municipal health centres by GPs, physicians specialising to be GPs, and doctors without any specialty. If specialised paediatric care is needed, children are referred to central or university hospitals. After-hours care is usually centralised into regional emergency clinics, and more complex cases are referred to secondary care, typically provided by hospital districts or university hospitals.

The private healthcare sector also offers services for the paediatric population, especially in urban areas. Private clinics may provide faster and direct access also to specialists (without referral). In the private sector, in addition to GPs and non-specialists, acute infections in children are also treated by specialists, particularly ENT specialists and paediatricians. There are also major differences in the types of patients and illnesses treated by specialists in the public vs. private sectors. For example, in the public sector, ENT specialists typically do not treat common respiratory infections. Paediatricians and physicians specialising to become paediatricians treat some common infections in public hospital emergency departments, but in the private sector, common infections are more frequently treated by paediatric specialists compared to the public sector.

**The use of private sector services in Finland**

Most families who use private care have voluntary private insurance, which covers part or all the cost. Some employers also provide private healthcare benefits for employees’ children. Based on the FinLapset study findings from the 2023 report, approximately 60% of infants (in 2020) and 58% of four-year-old children (in 2018) in Finland had a private health insurance policy. These figures come from large national surveys with thousands of respondents and are considered reliable estimates for children in those age groups. In absolute terms, according to data from the Finnish Financial Supervisory Authority, there were 462,000 children with a private health insurance in Finland as of June 2022. This represents a significant portion of the total child population in Finland, especially considering that the total number of children under 15 was around 860,000 in that same period.

According to the FinLapset 2023 report, about 28% of children aged 0–3 years in Finland used private healthcare services (specifically private doctor visits) in 2021. For the 1–3-year-old age group, the percentage was slightly higher at 32%. These figures are based on the Social Insurance Institution of Finland’s (Kela) reimbursement data, which tracks private doctor visits that were at least partially reimbursed by the national health insurance system (excluding dental care).

Children in urban and suburban municipalities were significantly more likely to visit private doctors than those in rural areas. In densely populated municipalities, access to private clinics is easier and more convenient, which contributes to higher usage. In rural areas, private healthcare services are less available or may require long travel distances, which limits their use. In the highest usage areas, 50%–60% of children in certain age groups visited a private doctor in 2021. In the lowest usage areas, less than 10% of children visited a private practice.

More about the FinLapset report online at https://thl.fi/tutkimus-ja-kehittaminen/tutkimukset-ja-hankkeet/finlapset-lasten-nuorten-ja-perheiden-terveys-hyvinvointi-ja-palvelut/finlapset-tutkimus
